# Chronic osteomyelitis at the base of coracoid process: an unusual location

**DOI:** 10.1093/jscr/rjaf312

**Published:** 2025-05-15

**Authors:** Feras Abuqweider, Abdullhaq Shaheen, Islam Bassam Atawna, Kinana Dababsa, Nadeen Sayarah, Eman AL-Najjar, Anwar Yousef Jabari

**Affiliations:** Faculty of Medicine, Palestine Polytechnic University, Hebron, Palestine; Orthopedic Department, Hilal Hospital, Hebron, Palestine; Faculty of Medicine, Palestine Polytechnic University, Hebron, Palestine; Orthopedic Department, Hilal Hospital, Hebron, Palestine; Faculty of Medicine, Palestine Polytechnic University, Hebron, Palestine; Faculty of Medicine, Palestine Polytechnic University, Hebron, Palestine; Faculty of Medicine, Palestine Polytechnic University, Hebron, Palestine; Faculty of Medicine, Palestine Polytechnic University, Hebron, Palestine; Faculty of Medicine, Palestine Polytechnic University, Hebron, Palestine

**Keywords:** chronic osteomyelitis, base of coracoid process, scapula, deltopectoral approach

## Abstract

Chronic osteomyelitis of the coracoid process is extremely rare with limited medical literature. This case reported a 17-year-old male who presented with persistent left shoulder pain and restricted range of motion for 2 months without a history of trauma. Imaging revealed an osteolytic lesion at the base of the coracoid process, confirmed by surgical biopsy. Initially, the patient underwent conservative management with non-steroidal anti-inflammatory drugs, but symptoms persisted, requiring surgical intervention involving decortication, biopsy, and fixation of the lesion using a screw. Histopathological analysis confirmed chronic osteomyelitis, and postoperative intravenous antibiotics for 3 weeks then symptom relief and restored shoulder function. This case highlights the diagnostic challenges for atypical presentation of chronic osteomyelitis in rare anatomical sites, emphasizing the importance of advanced imaging, prompt surgical intervention, and biopsy confirmation. Clinicians should consider osteomyelitis in the differential diagnosis of persistent shoulder pain, even in the absence of trauma or systemic signs of infection.

## Introduction

Osteomyelitis is an infection-related inflammatory bone disease that can be either acute or chronic [1]. However, it is a challenging condition characterized by persistent inflammation and infection of the bone. It most commonly affects long bones like the tibia or femur, but it is rare in flat bones such as the scapula [2], with unspecific symptoms, diagnosis and treatment are sometimes delayed [3], especially at the base of the coracoid process. The unusual location and nonspecific clinical presentation of chronic osteomyelitis in this region make it a diagnostic and therapeutic challenge for clinicians.

While a detailed clinical history, thorough examination, and appropriate imaging are crucial, histopathological confirmation through biopsy is essential for the definitive diagnosis of osteomyelitis [1]. The treatment of osteomyelitis involves targeted antibiotic therapy and often necessitates surgical intervention to eliminate infected and necrotic tissue [4].

In this report, we describe a challenging case of a 17-year-old male who presented with chronic osteomyelitis at the base of the coracoid process, a site rarely described in medical literature. The case highlights the diagnostic challenges and the role of imaging, surgical intervention, and management to prevent complications and restore function. This report aims to raise awareness of this rare presentation and provide insights into its clinical and surgical management.

## Case presentation

**Figure 1:**
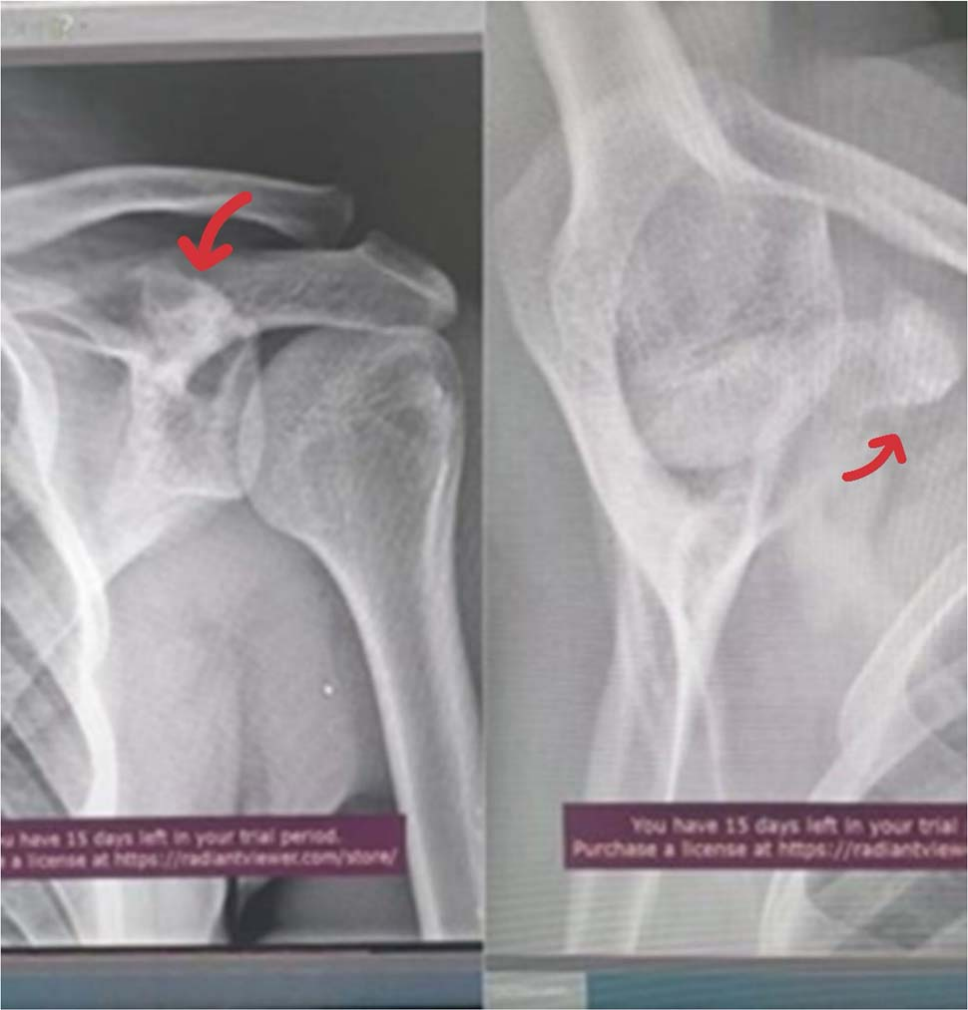
AP radiograph of the left shoulder shows a suspension lesion in the coracoid process of the scapula (indicated by arrow).

**Figure 2:**
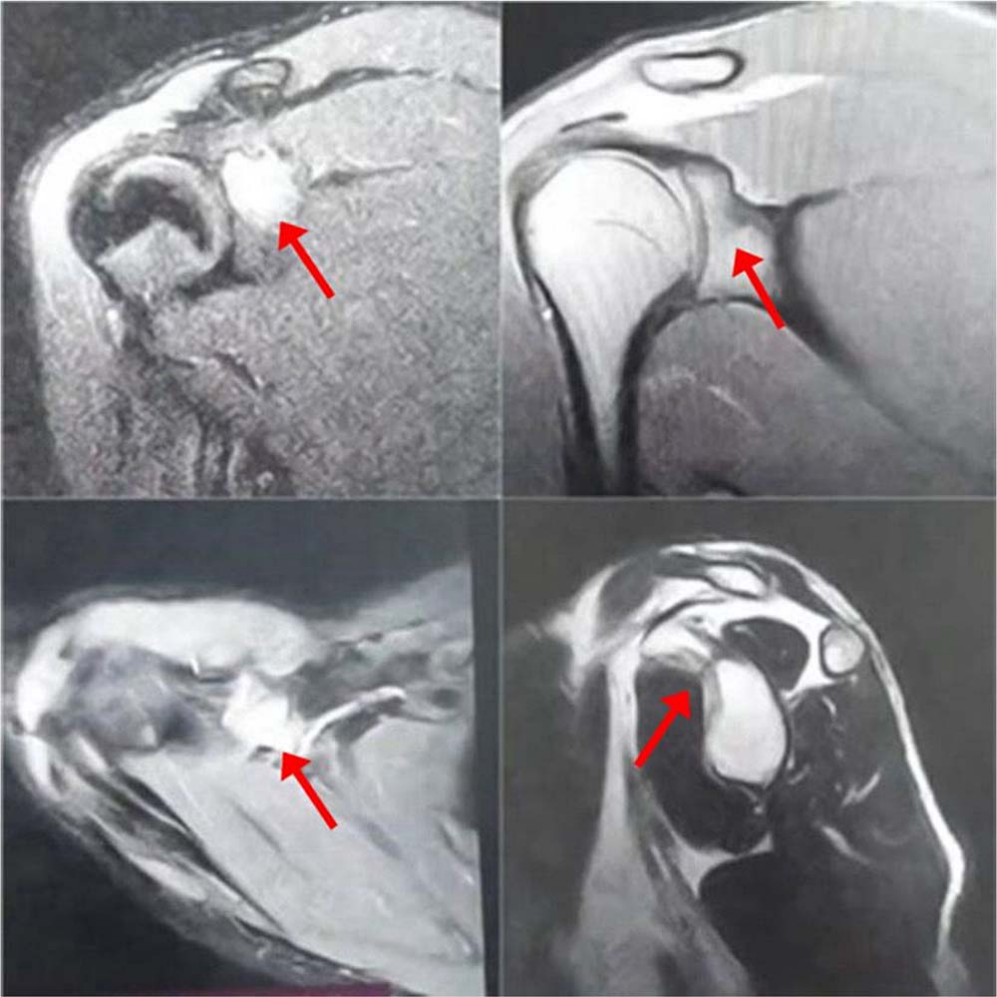
MRI without contrast of the left shoulder show a cortical and subcortical bony defect at the glenoid basecoracoid junction with hyperintense lesion measuring 8 × 7 mm (indicated by arrow), also intense bone marrow edema at the coracoid processes and the glenoid base-coracoid with surrounding fluid and edema extending to the coracoid-humeral space was seen.

A 17-year-old male presented with left shoulder pain and limited range of motion for 2 months, with no history of trauma or prior medical or surgical issues. On examination, there was tenderness localized to the coracoid process. Initial laboratory tests, including complete blood count (CBC), C-reactive protein (CRP), and erythrocyte sedimentation rate (ESR), all normal. Conservative management with rest and non-steroidal anti-inflammatory drugs (NSAIDs) was initiated, but there's no improvement. Initially, imaging with an X-ray revealed a suspicious lesion at the base of the coracoid process (Fig. 1), prompting further evaluation with magnetic resonance imaging (MRI) without contrast, which confirmed the lesion in the base of the coracoid with surrounding fluid and edema extending to the coracoid-humeral space (Fig. 2).

The differential diagnosis included chronic osteomyelitis, osteoid osteoma, eosinophilic granuloma, aneurysmal bone cyst, chondrosarcoma, osteosarcoma, and fibrous dysplasia.

**Figure 3:**
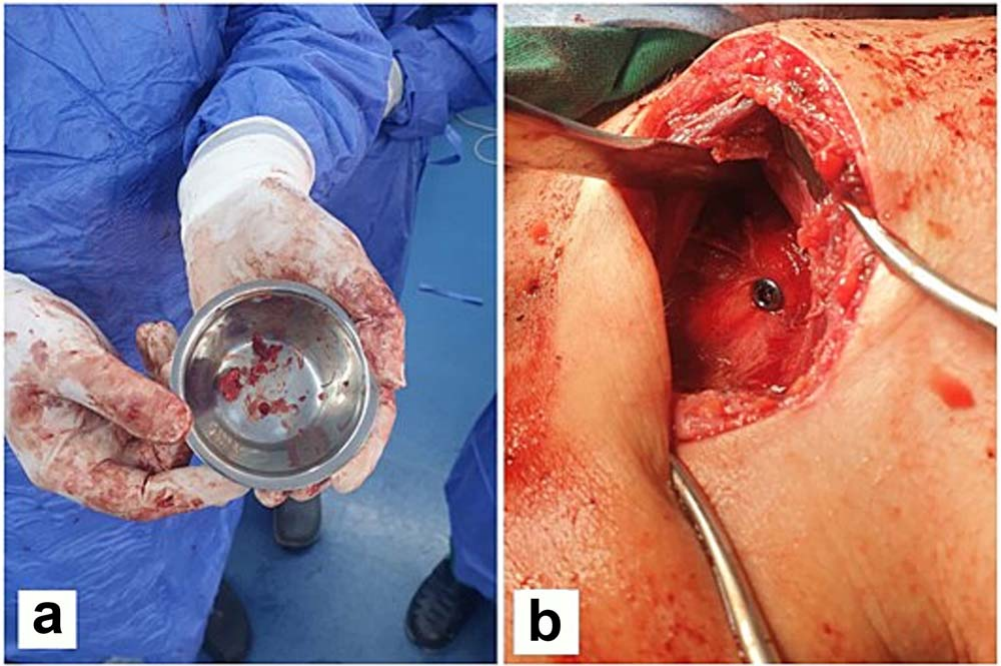
Multiple fragments of tan and bony tissue measuring in aggregate 2 × 1.5 cm taken from the base of the coracoid process (a). Intraoperatively, fixation of the coracoid process with a screw (b).

**Figure 4:**
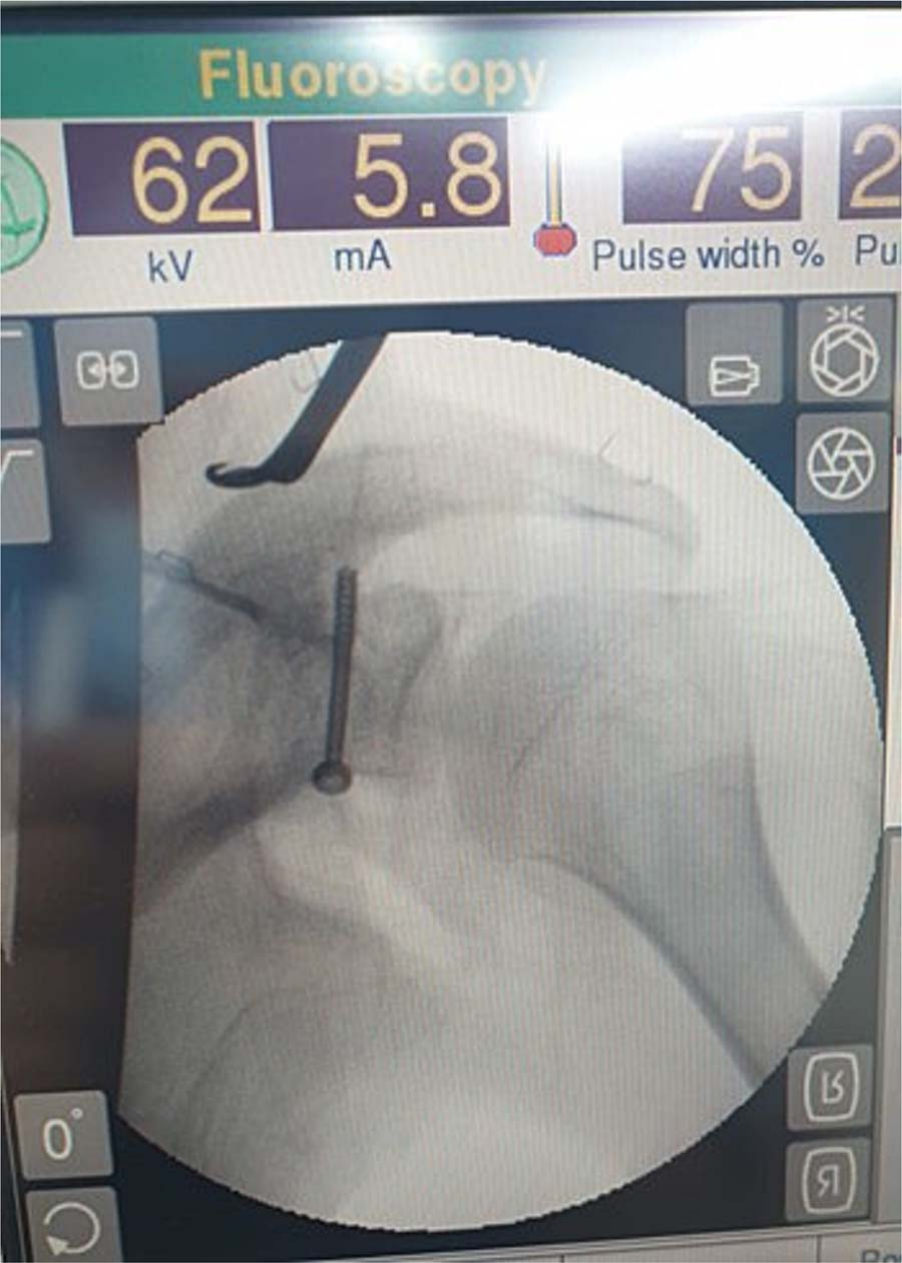
X-ray of the left shoulder post-operatively which shows proper placement of the screw.

The patient initially underwent conservative treatment with NSAIDs and rest, but symptoms did not improve. As a result, surgical intervention was planned using the deltopectoral approach. The procedure begins with the patient positioned in the beach chair position under general anesthesia. A longitudinal incision is made along the deltopectoral groove, and the cephalic vein is identified and retracted laterally with the deltoid. The deltoid is retracted laterally and the pectoralis major medially to expose the clavipectoral fascia, which is incised to access the coracoid process. Blunt dissection is used to expose the base of the coracoid while avoiding neurovascular structures. Decortication of the coracoid base is performed, followed by biopsy of the lesion (Fig. 3a) and fixation of the coracoid process with a screw (Fig. 3b). An X-ray was performed postoperatively to confirm proper placement of the screw (Fig. 4).

Postoperatively, the biopsy result confirmed chronic osteomyelitis, so the patient was started on intravenous antibiotics—Augmentin and clindamycin—for 3 weeks. The patient also underwent two postoperative physiotherapy sessions to improve range of motion. Following treatment, the patient demonstrated a full recovery, with restored range of motion, complete resolution of pain, and high satisfaction with the outcome. The duration of follow-up was ~6–7 months, during which no recurrence or complications were observed.

## Discussion

Osteomyelitis is an infection-related inflammatory bone disease [1]. Lew and Waldvogel were the first to classify osteomyelitis based on the duration and mechanism of infection [5]. The categorization of osteomyelitis as acute or chronic is based on the histopathological findings rather than the duration of illness [5]. Acute osteomyelitis is characterized by infection before the formation of sequestra and typically occurs within 2 weeks of disease onset. Symptoms of acute osteomyelitis include localized pain, swelling, and fever [1, 5]. Symptoms of chronic osteomyelitis may manifest over a prolonged period, typically >2 weeks. Similar to acute osteomyelitis, patients may exhibit erythema, discomfort, and edema at the infection site; however, fever and other constitutional symptoms are less common. Localized bone loss and the reactive bony encasement of the sequestrum, known as an involucrum, are other common characteristics of chronic osteomyelitis [6].

The humerus, tibia, femur, maxilla, vertebrae, and maxillary bones are the most commonly affected sites in osteomyelitis. In children, long bones are more frequently involved due to the highly vascular nature of their metaphysis. In contrast, the involvement of short, flat bones, such as the scapulae, is rare [2].

A systematic review by Koubaa *et al.* [3] analysed reported cases of osteomyelitis over 10 years and identified only four cases involving the scapula, representing an incidence of 2.6%.

In this specific case, osteomyelitis occurred at the base of the coracoid process of the scapula, which is an uncommon site for infection. The coracoid process is rarely affected due to its limited vascularity and relative isolation from direct trauma or soft tissue infections. A clinical suspicion of osteomyelitis is essential to initiate a medical evaluation and is influenced by various factors such as the duration of the condition and the specific bone involved [7, 8]. Particularly in non-specific areas, the diagnosis of osteomyelitis involves assessing a combination of clinical signs and symptoms, conducting laboratory investigations, using imaging modalities, and performing histopathological examinations [9].

Laboratory tests such as ESR and CRP are important [10], as leukocytosis may be present in acute osteomyelitis but may be absent in chronic osteomyelitis [1]. Therefore, these tests are not specific, and further investigations, such as imaging and tissue biopsy, should be conducted [9]. Clinically, the more effective studies are X-ray, MRI, computed tomography (CT), and technetium 99 bone scintigraphy [1].

In chronic osteomyelitis, an X-ray may show the thickness and irregular bone [11], but there's a limitation may have a delay about 14 days before the appearance of the changes, which leads to the use of more images like MRI and bone scintigraphy [6]. MRI is considered the most reliable and effective method for diagnosing osteomyelitis [10]. CT is a useful tool for evaluating osteomyelitis and complementing other imaging techniques [12]. However, histological analysis of bone, soft tissue, and sequestra obtained during biopsy or surgical debridement is essential to confirm the diagnosis [9].

The treatment of osteomyelitis, irrespective of its location, begins with targeted antibiotic therapy and immobilization of the affected area. In severe cases, surgical intervention is needed to remove infected or necrotic tissue [10], during this procedure it is crucial to confirm that all unwanted tissue has been excised. Pathology findings often inform whether further debridement is needed [5].

## Conclusion

Chronic osteomyelitis of the coracoid process, though actually rare, should be considered in the differential diagnosis of persistent shoulder pain, particularly in young patients without a history of trauma. Early imaging, including MRI, plays a crucial role in identifying the lesion. However, the definitive diagnosis required histopathological confirmation through biopsy. Surgical intervention, including decortication and fixation, followed by appropriate antibiotic therapy, leads to resolution of symptoms and restoration of function. This case confirms the need for thorough preoperative evaluation and the role of biopsy in managing complex and atypical presentations of osteomyelitis.

## Data Availability

The data used to support the findings of this study are included in the article.
